# Impact of Incorporating Shiitake Mushrooms (*Lentinula edodes*) on Microbial Community and Flavor Volatiles in Traditional Jiuqu

**DOI:** 10.3390/foods13071019

**Published:** 2024-03-26

**Authors:** Jingzhang Geng, Siqiao He, Shanshan Zhang, Honglei Tian, Wengang Jin

**Affiliations:** 1 National Key Laboratory of Biological Resource and Ecological Protection Jointly Built by the Province and Ministry, Shaanxi University of Technology, Hanzhong 723001, China; gengjingzhang@163.com (J.G.); sqhe0112@163.com (S.H.); zshanshan2024@163.com (S.Z.); thl0993@sina.com (H.T.); 2 School of Life Science and Technology, Xi’an Jiaotong University, Xianning West Road, Xi’an 710049, China; 3 Collaborative Innovation Center of Bio-Resource in Qin-Ba Mountain Area, Key Laboratory of Bio-Resources of Shaanxi Province, Shaanxi University of Technology, Hanzhong 723001, China

**Keywords:** shiitake mushroom, Jiuqu, microbial community, volatile organic compounds

## Abstract

Jiuqu is one of the important raw materials for brewing Chinese rice wine (Huangjiu), often known as the “bone of wine”. In this study, the microbial community and flavor substances of Jiuqu made with different amounts of shiitake mushroom (*Lentinula edodes*) were investigated through high-throughput sequencing technology and headspace gas chromatography–ion migration spectroscopy (HS-GC-IMS), using traditional wheat yeast as a control. The results showed that 1593 genera and 5507 species were identified among the four types of yeast, with *Aspergillus* and *Paecilomyces* being the most dominant microorganisms at the genus level. Carbohydrate, coenzyme, and amino acid metabolism may be the main metabolic processes of the dominant microorganisms in Jiuqu. In terms of flavor, a total of 79 volatile substance monomers and some dimers were detected from four types of Jiuqu raw materials, with the main substances being 12 aldehydes, 19 ketones, 13 alcohols, 19 esters, 4 olefins, 1 acid, 3 ethers, 4 furans, 1 pyrazine, 1 pyridine, 1 triethylamine, and 1 thiazole. The correlation results indicate that *Aspergillus*, *Lactobacillus*, and *Vibrio* correlate significantly with the volatile flavor compounds unique to shiitake mushrooms and also have a positive effect on alcohol, esters, and furans. These results could shed light on the selection of *Lentinula edodes* as a fermentation starter for Huangjiu in the Qinba Mountain area.

## 1. Introduction

Huangjiu is a traditional Chinese brewed rice wine, with a history dating back more than 5000 years. Along with beer and wine, it is known as one of the three ancient liquors in the world [1,2]. Due to its rich nutritional value and unique flavor, it is also known as the “ancestor of wine and liquid cake” and has deeply fascinated its consumers. There are many production areas of Huangjiu in China, which are widely distributed and can be divided into southern Huangjiu and northern Huangjiu areas. The traditional process usually uses glutinous rice, millet, corn, sorghum, and other grains rich in starch as raw materials [3,4], and Jiuqu as a saccharification and fermentation agent. It is brewed through processes such as soaking rice, steaming rice, cooling, dropping jars, mixing Jiuqu, fermentation, pressing, clarification, filtration, sterilization, etc. [5]. As advantageous characteristics, the edible mushroom species in the Qinba Mountain area, shiitake mushrooms (*Lentinula edodes*), have edible value in reducing cholesterol, preventing arteriosclerosis, promoting children’s growth and development, enhancing human immune function, and preventing and treating cancer [6]. Shiitake mushroom Huangjiu is made from *Lentinula edodes* through traditional brewing techniques [7,8].

The traditional brewing process of Huangjiu is similar to and a type of open fermentation. Microorganisms in the environment will participate in the fermentation process together with the main brewing microorganisms, thereby affecting the formation of flavor substances in Chinese Huangjiu [9]. Jiuqu is one of the most important raw materials for brewing traditional Chinese Huangjiu, often known as the “bone of wine” [10]. Liquor yeast contains a wide variety of microorganisms, including both bacteria and fungi such as yeast and mold. Fungi, yeast, and bacteria are believed to produce a large amount of enzymes for cellular metabolism and subsequent small molecule generation, which contribute to the formation of flavor in the final product [11]. The various metabolites of microorganisms play a very important role in the flavor, taste, quality, and function of wine. Therefore, the diversity analysis of yeast microorganisms has important practical significance in stabilizing and improving the quality of Chinese Huangjiu [12].

In recent years, many scholars have begun to research Jiuqu, and most of their research methods revolve around high-throughput sequencing technology, gas chromatography–mass spectrometry (GC-MS), and so on. Cai et al. [13] performed high-throughput sequencing on eight starter culture samples and identified 10 genera of yeast and mold and 11 genera of bacteria; a fungal diversity analysis showed that there were significant differences in the fungal components in the starter samples and that the microbiota of the starter culture was mainly *Rhizopus* spp., indicating that it played an important role in starch hydrolysis during the brewing process of sweet Huangjiu. Li Bin et al. [14] used high-throughput sequencing to analyze the microbial diversity of red yeast rice and large yeast and small yeast rice. The results showed that the microbial community diversity of Daqu was the highest, while that of Hongyeast was the lowest, and that the microbial community structure difference between the two was the largest. Therefore, high-throughput sequencing technology has the advantages of a high throughput, low cost, simple operation, and accurate results and can accurately and quickly analyze the complex microbial community structure in samples, making it the best method for the analysis of the complex microbial diversity in Huangjiu samples [15]. Wang et al. [16] analyzed the microbial community structure in Jiuqu based on high-throughput sequencing technology, combined it with headspace solid-phase microextraction–gas chromatography–mass spectrometry (HS-SPME-GC-MS) technology to detect its volatile flavor compounds, and explored the correlation. The results showed that a total of 10 dominant bacterial genera, 7 dominant fungal genera and 22 volatile flavor compounds were detected in their three Jiuqu samples, and some microorganisms were correlated with the volatile flavor compounds [17]. However, there are few studies on the annotation of the microbial gene functions of Jiuqu, and these cannot better explain the effect of the metabolism of the microbial community on flavor during the fermentation process. Therefore, an in-depth exploration of the microbial community structure and gene function annotation of Jiuqu could help to us analyze the relationship between Jiuqu and flavor, reveal the flavor production mechanisms in the process of Jiuqu making, and would also have certain predictive significance for subsequent brewing.

Compared with the mainstream GC-MS, gas chromatography–ion mobility spectrometry (GC-IMS) has become a popular method for the separation and detection of flavor components in recent years, with the comprehensive advantages of the high separation efficiency and high sensitivity of ion mobility spectrometry and the advantages of simple operation and high efficiency. It is increasingly used in the field of food flavor detection. At present, the microbial community structures and flavor characteristics of Jiuqu, based on high-throughput sequencing combined with HS-SPME-GC-MS, have been reported [17,18], whereas the microbial diversity and flavor profiles of Jiuqu, especially with *Lentinula edodes’* incorporation, based on high-throughput sequencing combined with gas chromatography–ion mobility spectrometry (GC-IMS), are still unknown. In this regard, a correlation analysis was performed and the effects of the incorporation of *Lentinula edodes* on the microbial communities and flavor substances of Jiuqu in the Qinba Mountain area were characterized in this study, which might shed light on the innovation of traditional Jiuqu production and the utilization of the abundant *Lentinula edodes* resources in the Qinba Mountain area (Hanzhong, China).

## 2. Materials and Methods

### 2.1. Materials and Reagents

Jiuqu (traditional wheat Qu)(Shaanxi Changsheng Liquor Co., Ltd., Hanzhong, China); shiitake mushrooms and fragrant rice (Taoxindao Supermarket Co., Ltd., Hanzhong, China. 2-butanone, 2-pentanone, 2-hexanone, 2-heptanone, 2-octanone, and 2-nonanone (Sinopharm Chemical Reagent Co., Ltd., Beijing, China). Other chemical reagents were of analytical grade (Sangon Bioengineering Co., Ltd. Shanghai, China).

### 2.2. Preparation of Shiitake Mushroom Jiuqu

For shiitake mushroom (*Lentinula edodes*) and wheat Jiuqu, select high-quality wheat produced that year, with full grains, uniform particles, and thin skins; put the wheat and *Lentinula edodes* powder (2.5%, 3%, and 5% of the mass fraction, respectively) into a Jiuqu mixer, add 19–22% water, and stir evenly. The mixing time is 5 min, otherwise it will absorb too much water and it will be difficult to shape. After mixing the Jiuqu, place the Jiuqu pieces (25 cm × 15 cm × 3 cm), with a distance of 5–8 cm between each piece, close to doors and windows, and keep them warm for cultivation. Keep the temperature at 30–40 °C for 1–2 days to allow microorganisms to multiply in large quantities. Then, adjust the temperature to 40–50 °C for the next 2–3 days. After that, the Jiuqu enters a period of moisture release and aroma production; the water evaporates and the product’s temperature gradually decreases. After about 30–40 days of cultivation in the Jiuqu room, the finished Jiuqu are obtained. According to different amounts of shiitake mushrooms (Xiang Gu in Chinese) used, the finished Jiuqu were labeled as XG100 (with 2.5% *Lentinula edodes* powder), XG120 (with 3% *Lentinula edodes* powder), and XG200 (with 5% *Lentinula edodes* powder), respectively. Traditional wheat Jiuqu (Pu Tong Mai qu in Chinese, PTMQ) can be prepared using the same method as above, but the raw materials should not include *Lentinula edodes* powder.

Sample preparation: Weigh 200 mg of the sample, put it into a sterilized centrifuge tube (2 mL), add 1 mL of 70% ethanol, shake to mix, and centrifuge at 10,560× *g* in an HT165R Benchtop High Speed Refrigerated Centrifuge (Hunan Xiangyi Laboratory Instrument Development Co. Ltd., Changsha, China) for 3 min at room temperature, and discard the upper liquid. Add 1 × PBS solution, shake and mix, centrifuge at 10,560× *g* at room temperature for 3 min again, and discard the supernatant liquid. Invert the 2 mL centrifuge tube on absorbent paper for 1 min until no liquid flows out. Place the sample tube into an oven at 55 °C for 10 min.

### 2.3. DNA Extraction, Library Construction, and Quality Control

The pretreated samples were extracted according to the method described in the Omega manual [19]. DNA was broken down into approximately 500 bp fragments using a Covaris220 ultrasonic disruptor, purified using Hieff NGS™ DNA Selection Beads DNA reagents, and amplified using Hieff NGS^®^ MaxUp(Shanghai Yisheng Biotechnology Co., Ltd., Shanghai, China). DNA quality was assessed using Fastp (version 0.36) as follows: 1. remove adapters in sequence; 2. remove low-quality bases (Q < 20) from 3′ to 5′ ends and use the sliding window method to remove bases with tails of less than 20 DNA reads to find the base value (window size is 4 bp); 3. find the overlapping parts of the DNA reads and appropriately correct the inconsistent bases in the interval; 4. remove DNA reads less than 35 nt in length.

### 2.4. Assembling and Cartooning

First, use Megahit (version 1.2.9) to perform multi-sample hybrid splicing to obtain preliminary splicing sequences and then use bowite2 (version 2.1.0) to obtain clear DNA reads. Control the spliced results, extract unspliced DNA reads, and then use SPAdes (Version 3.13) to splice again to obtain low-abundance allele groups; MetaWRAP (Version 1.3.2) was used to sequentially perform the Bin classification, Bin purification, Bin quantification, Bin recombination, and Bin identification of the DNA reads.

### 2.5. Gene Prediction and Non-Redundant Genome Construction

Use Prodigal (version 2.60) to predict the ORF of the splicing results, select genes with a length greater than or equal to 100 bp, and translate them into nucleic acid sequences. For the gene prediction results of each sample, use CD-HIT (version 2.60) to remove duplications to obtain a non-redundant gene set; use Salmon (version 1.5.0) to construct a specific index of the non-redundant genome, use the biphasic algorithm and method of building a bias model to accurately quantify the gene abundance in each sample, and use the gene length information to calculate gene abundance.

### 2.6. Illumina HiSeq 2000 Platform Sequencing

The DNA that passed the quality control was sequenced on a machine (completed by the Illumina HiSeq 2000 sequencing platform (Shanghai Sangon Bioengineering Co., Ltd. Shanghai, China). For species and functional annotation, use DIAMOND (version 0.8.20) to compare the gene set with the KEGG, CAZy, and SEED databases to obtain the species annotation information and functional annotation information of the genes. Filtering conditions: E value < 10^−5^. Score > 60.

### 2.7. Quantification of Flavor Volatiles by GC-IMS

Flavor volatiles were analyzed using an Agilent 8890 GC system (Agilent Technologies, Palo Alto, CA, USA) and an IMS instrument (FlavourSpec^®^, Gesellschaft für Analytische Sensorsysteme mbH, Dortmund, Germany). We weighed 2.0 g of the different Jiuqu samples, placed them in 20 mL headspace vials, and added 20 μL of internal standard (2-butanone, 2-pentanone, 2-hexanone, 2-heptanone, 2-octanone, and 2-nonanone, 10 ppm), and made three replicates of each, respectively. The following instrumental parameters were modified from Jin et al. [18]. Headspace sampling conditions: headspace incubation temperature—60 °C; incubation time—10 min; heating method—oscillating heating; incubation speed—500 rpm, headspace needle temperature—85 °C; injection volume—100 μL; no Split mode; high-purity nitrogen (purity ≥ 99.999%) to push and clean the headspace needle; cleaning time—20 min. Chromatographic conditions: Chromatographic column MXT-WAX (30 m × 0.53 mm, 1 μm); column temperature—60 °C; carrier gas—high-purity nitrogen (purity ≥ 99.999%); carrier gas program—2 mL/min, lasting 2 min, and then increase to 100 mL/min within 18 min, and maintain at 100 mL/min until 40 min. Ion mobility spectrum conditions: drift tube length—10 cm; linear voltage in the tube—400 V/cm; drift tube temperature—40 °C; drift gas—high-purity nitrogen (purity ≥ 99.999%); flow rate—150 mL/min; IMS detector temperature—45 °C.

### 2.8. Statistical Analysis

All data were expressed as the mean ± standard deviation (*n* = 3). The characterization of VOCs was performed using the NIST 2014 and IMS databases. The multiple comparisons were analyzed through a one-way analysis of variance and Duncan’s multiple tests using the SPSS 22.0 Software Package (SPSS, Inc., Chicago, IL, USA). A level of *p* < 0.05 was considered as significant. The PCA and correlation heatmap were visualized through an online R package for data visualization (https://cloud.metware.cn, accessed on 20 February 2024).

## 3. Results and Discussion

### 3.1. Alpha Diversity Analysis

Our library reflects the coverage of each sample in the comparison library. The higher the value, the lower the probability that the sequence in the sample is not detected. The library coverage rates for the samples in this experiment were all greater than 99%, indicating that the sequencing results could represent the true situation of the samples. The Alpha index can reflect the richness and evenness of the microbial species in a sample [20]. The larger the Chao1 and ACE indices, the higher the species richness. The larger the Shannon and Invsimpson indices, the better the species evenness. As shown in Table 1, the Chao1, ACE, Shannon and InvSimpson indices are all ordered as follows: XG100 > XG120 > XG200 > PTMQ, indicating that species richness and evenness are both in the following order: XG100 > XG120 > XG200 > PTMQ.

The above results show that introducing shiitake mushrooms as a raw material to make Jiuqu has a great impact on the composition of the microbial community in the finished Jiuqu, which is mainly manifested in their improvement of the diversity and uniformity of the Jiuqu microbial community. Our previous study has shown that adding shiitake mushrooms during the making of Jiuqu is beneficial to the growth of the microorganisms in the finished Jiuqu. It can improve the saccharification and fermentation capabilities of the Jiuqu by providing nutritional factors for beneficial bacteria and inhibiting the growth of miscellaneous bacteria.

The main active ingredients in shiitake mushrooms are lentinan, purine, nuclear acid, and vitamins [6,7], which are plant-derived prebiotics that can inhibit miscellaneous bacteria and promote the growth of various functional microorganisms in wheat Jiuqu. As a result, the growth and reproductive activities of multiple functional microorganisms in Jiuqu are strengthened, and its diversity and uniformity are improved. When an appropriate amount of the raw material of shiitake mushrooms is added (2.5%, XG100), it will have a great impact on the diversity of the microbial community in Jiuqu and increase the total number of microbial colonies. However, when too many mushrooms (above 3%, XG120 and XG200) are added, they will not only reduce the total number of microorganisms in the Jiuqu, but also significantly reduce the diversity of the microorganism community in the finished Jiuqu. It is speculated that the impact of the addition of shiitake mushrooms on the total number and diversity of the microbial community may be due to the excessive lentinan in shiitake mushrooms, which has a certain inhibitory effect on the microorganisms in finished Jiuqu [6,19].

### 3.2. Analysis of the Differences in Jiuqu with Different Amounts of Shiitake Mushrooms Added

**Figure 1:**
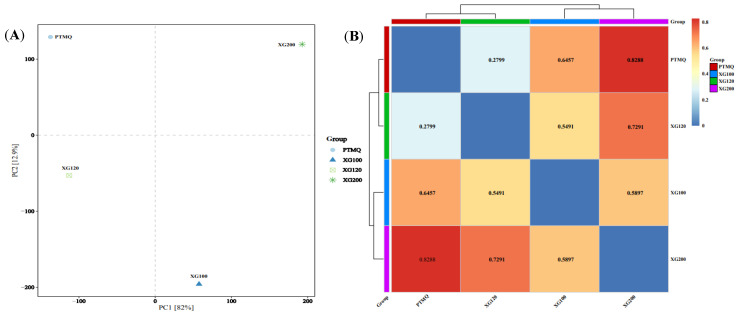
The PCA score plot (**A**) and heat map of the distances (**B**) of different types of Jiuqu at the genus classification level.

Principal component analysis (PCA) forms a dimensionality reduction effect by retaining the components that contribute the most to a sample’s characteristics and can intuitively display the differences between samples [21]. As shown in Figure 1A, the first and second principal coordinates can contribute 82% and 12.9% of the sample’s information, respectively, and can effectively reflect the differences between samples. The four types of Jiuqu are completely separated in the PCA and have strong differences. Among them, XG100 is the most distant and has the greatest difference from PTMQ, XG120, and XG200, probably because of its low level of shiitake mushrooms; PTMQ and XG120 are closer and have smaller differences. The distance heat map between samples at the genus classification level is shown in Figure 1B. It can be more intuitively judged that the community composition of PTMQ and XG120 is similar; however, XG100 and XG200 are clustered together horizontally and vertically, with a numerical difference of 0.5897, indicating that there are large differences in the microbial communities of these two types of Jiuqu that are clustered together. This result is consistent with the PCA results.

### 3.3. Species Composition Analysis

**Figure 2:**
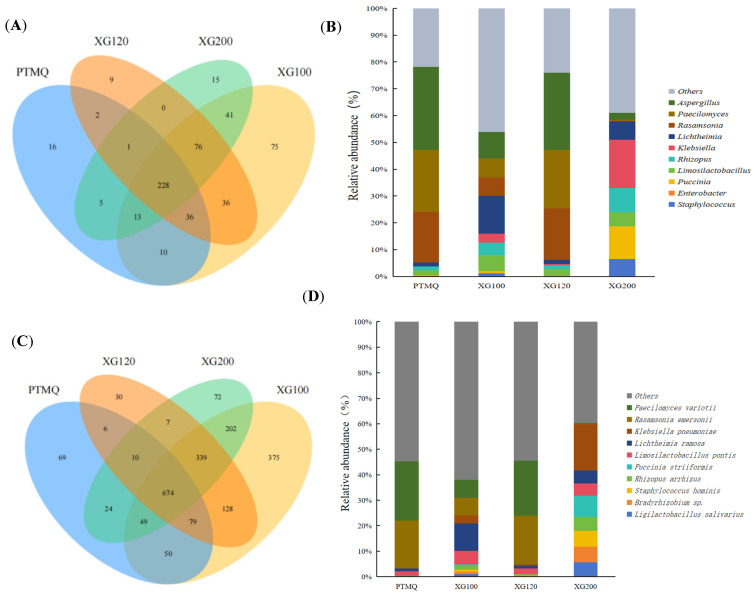
Venn diagram and microbial community stacked histogram in Jiuqu with different amounts of shiitake mushrooms added. (**A**,**C**) denote Venn diagrams at the genus and species levels, respectively, while (**B**,**D**) denote their microbial community stacked histograms.

The dominant bacterial flora play an important role in the microbial structure of Jiuqu. As shown in Figure 2A, on the genus level, a total of 1593 genera of microorganisms were identified in the four types of Jiuqu. Among them, PTMQ was annotated a with total of 311 genera, XG100 was annotated with 515, XG120 was annotated with 388, and XG200 was annotated with 379, indicating that XG100 has the highest diversity of genera, while PTMQ has the lowest.

The genera with the highest proportions of the top ten dominant microorganisms in the four Jiuqu samples were Aspergillus, Paecilomyces, Rasamsonia, Lichtheimia, Klebsiella, Rhizopus, Limosilactobacillus, Puccinia, Enterobacter, and Staphylococcus (Figure 2B). The bacterial genera with the highest proportions in PTMQ and XG120 were Aspergillus, Paecilomyces, and Rasamsonia. For PTMQ, their abundance proportions were 31.03%, 23.19%, and 18.81%, while their abundance proportions in XG120 were 28.87%, 21.77%, and 19.19%, respectively; the dominant microbial genera in XG100 were mainly Lichtheimia (14.02%), Aspergillus (9.83%), and Paecilomyces (7.11%); the dominant microbial genera in XG200 were mainly Klebsiella (18.04%), Puccinia (12.26%), and Rhizopus (9.04%). 

It can be seen that the genera *Aspergillus* and *Paecilomyces* are the most dominant microorganisms, with high relative abundance at the genus level, and are distributed across the four Jiuqu samples (Figure 2B). Studies have shown that *Aspergillus* mainly provides saccharification power, liquefaction power, various protein hydrolysis capabilities, partial acid and ester production capabilities for the brewing process, and makes important contributions to fermentation and flavor formation [22,23]. The genera of XG100 are relatively evenly distributed compared to the other three Jiuqu samples, which is also consistent with previous species diversity analysis results. As found in our previous work [19], stable microbial fermentation can improve the flavor and quality of the finished wine. 

At the species level, the microbial community structure of the four types of Jiuqu was analyzed, and a total of 5507 species of microorganisms were identified (Figure 2C). Among them, PTMQ was annotated with a total of 961 species, XG100 was annotated with 1896, XG120 was annotated with 1273, and XG200 was annotated with 1377. The four types of Jiuqu have 674 identical microorganisms, accounting for 70.13%, 35.55%, 52.95%, and 48.95% of their total number of annotated species, respectively. Judging from the proportion of similar species, the species composition of XG100 is quite different from that of the other three types of Jiuqu, while PTMQ and XG120 have a relatively high degree of similarity in their species composition. From a macro perspective, XG100 and XG200 are more evenly distributed than PTMQ and XG 120. From a micro view, the top 10 microorganisms found in the four Jiuqu are *Paecilomyces variotii*, *Rasamsonia emersonii*, *Klebsiella pneumoniae*, *Lichtheimia ramosa*, *Limosilactobacillus pontis*, *Puccinia striiformis*, *Rhizopus arrhizus*, *Staphylococcus hominis*, *Bradyrhizobium*, and *Ligilactobacillus salivarius* (Figure 2D).

Among them, the bacterial species that were more highly abundant in PTMQ and XG120 were *Paecilomyces variotii* and *Rasamsonia emersonii.* Their abundance proportions in PTMQ were 23.12% and 18.81%, while in XG120 they were 21.69% and 19.19%, respectively, (Figure 2D), which is consistent with the above-mentioned species composition of PTMQ and XG120, which have a high degree of similarity. Studies have shown that *Paecilomyces variotii* has high glucoamylase activity, can decompose starch in raw materials, and ferment to produce a variety of flavor substances and flavor precursors [9]. The bacterial species most abundant in XG100 Jiuqu was *Lichtheimia ramosa*, and this Jiuqu has the highest abundance of the species compared to the other three kinds of Jiuqu, with an abundance of 10.73%. The bacterial species most abundant in XG200 Jiuqu is *Klebsiella pneumoniae*. Its abundance can reach 17.98%.

Studies have shown that *Lichtheimia ramosa* has the ability to produce α-amylase, glucanase, xylanase, cellulase, protease, and glucoamylase [24,25]. *Lichtheimia ramosa* can produce various flavor substances, such as acetic acid, ethanol, phenethyl alcohol, and ethyl acetate [26]. Moreover, the mixture of *Lichtheimia ramosa* and Jiuqu can increase the diacetyl content of the base wine, increase its aroma, and increase the wine yield and quality of the product [27]. Therefore, XG100 might possess more advantages for brewing Huangjiu, because of the combined work between *Lichtheimia ramosa* and Jiuqu towards an improved aroma and yield. At the same time, alcohol production is positively correlated with *Klebsiella pneumoniae* [28].

### 3.4. Microbial Gene Function Annotation

#### 3.4.1. KEGG Function Annotation Analysis

**Figure 3:**
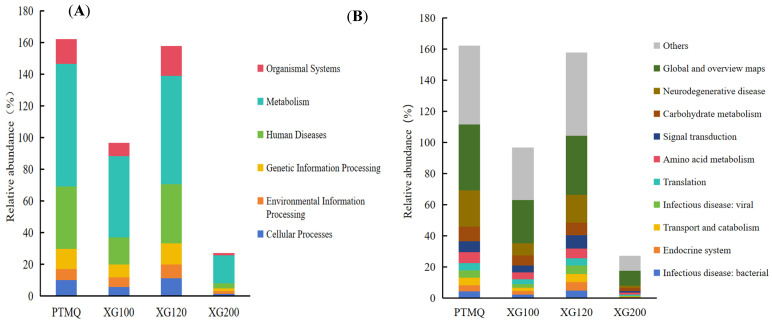
The KEGG first level (**A**) and second level (**B**) abundance of the functional genes of Jiuqu with different amounts of shiitake mushrooms added.

The KEGG (Kyoto Encyclopedia of Genes and Genomes) is a well-established database of biological systems, which includes information on genomes, chemical substances, and system functions. As shown in Figure 3A, XG200 has the lowest abundance of total functional genes among the six basic modules of the KEGG’s first-level functional annotation (biological systems, metabolism, human diseases, genetic information processing, environmental information processing, and cellular processes), indicating that the degree of microbial metabolic activity in this Jiuqu is relatively weak and that it may not be enough to produce sufficient extracellular enzymes and other functional substances to provide subsequent fermentation, which may have a certain inhibitory effect on the flavor and quality of the finished liquor. From the microscopic analysis, the metabolic module was the most dominant function of the four (its abundance accounted for 17% of the total), which provided a basis for the formation of the flavor of Huangjiu. Among the Jiuqu samples, the gene abundance of PTMQ accounted for its relatively high proportion of the metabolic function module, reaching 77.22%, followed by XG100 and XG120, while XG200 accounted for the lowest proportion. The metabolic function is the basis of microbial life activities, including lipid metabolism and protein metabolism, etc., which can decompose and consume the nutrients in raw materials for the mushrooms’ own growth and reproduction, and at the same time produces new secondary metabolites (Figure 3A), which have an important impact on the overall quality of the finished wine.

However, excessive metabolic activities will further deplete the nutrients in the brewing process, reduce the content of total sugars, amino acids, and functional polysaccharides in the finished wine, and also reduce the diversity and richness of the wine’s flavor compounds due to the reduction of its flavor precursors, thereby reducing the overall quality of the finished wine [29]. Therefore, XG100 has the potential to balance its microbial growth and body quality. Compared with the other three types of Jiuqu, the relative microbial content of XG100 has a higher uniformity of 8.24% compared to 7.95%, 6.13%, and 5.74%, respectively, which is conducive to the formation of a stable microbial fermentation system in the process of fermenting multiple strains, making it difficult to cause the excessive abundance of certain species that might seize the living space and resources of other beneficial bacteria, thus causing incomplete fermentation.

The secondary functions of the KEGG analysis include carbohydrate metabolism, amino acid metabolism, and gene classification modules such as transport and catabolism. As shown in Figure 3B, PTMQ, XG100, XG120, and XG200 had a high abundance of genes, with proportions of 42.4%, 27.81%, 37.67%, and 9.41%, in the metabolic pathway, respectively, which is related to starch and sucrose metabolism, fructose and mannose metabolism, and acid metabolism, which are important metabolic pathways in the fermentation process. PTMQ and XG120 contained relatively high proportions of 23.33% and 18.03%, respectively, while XG100 and XG200 contained relatively low proportions of 7.76% and 1.42%, respectively, which may be related to their low abundance of conditionally pathogenic bacteria, as noted above. In addition, XG100 has higher uniformity of 6.52%, 4.35%, 4.32% and 3.21% in the gene modules of carbohydrate metabolism, signal transduction, amino acid metabolism, and translation, respectively (Figure 3B). It not only ensures a number of genes present related to carbohydrate metabolism, but also will not cause a certain property of the wine to be too strong due to the lack of other related genes, resulting in the problem of unbalanced wine quality. Carbohydrate metabolism and amino acid metabolism are the metabolic basis of the flavor substances affecting Huangjiu, and they are also important metabolic pathways in Huangjiu starter cultures [30]. A large number of studies have shown that, in the formation of the flavor of fermented wine, reducing sugars and amino acids can form a series of flavor compounds, such as pyrazine, furan, aldehyde, and phenols, which make important contributions to the flavor of fermented wine [31].

#### 3.4.2. CAZy and SEED Function Annotation Analysis

CAZy is a carbohydrate-active enzyme database [32,33], specializing in the synthesis and decomposition of complex carbohydrates and glycocomplex enzymes, which can be divided into six major protein families according to the similarity of the amino acid sequences in the protein domains, including glycosyl transferases (GTs), glycoside hydrolases (GHs), carbohydrate esterases (CEs), oxidoreductases (Auxiliary Activities, AAs), Polysaccharide lyases (PLs) and Carbohydrate-Binding Modules (CBMs).

**Figure 4:**
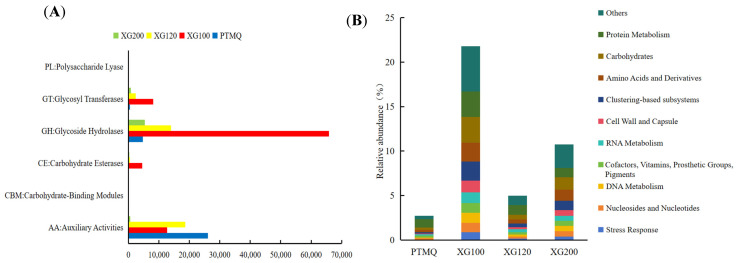
The abundance of genes according to the CAzy annotation (**A**) and SEED function annotation (**B**) of Jiuqu with different amounts of shiitake mushrooms added.

As shown in Figure 4A, the number of GHs in XG100 was much larger than that in PTMQ, XG120, and XG200, reaching 65843, accounting for 73.1% of the total number of GHs in the sample. Glycoside hydrolase can hydrolyze or rearrange glycosidic bonds [34], which have the function of decomposing carbohydrates, such as starch and cellulose. The higher the abundance of glycoside hydrolase genes, the faster the decomposition of carbohydrates, that is, the faster the saccharification rate at the beginning of fermentation, which is conducive to the metabolism of yeast to ethanol. Therefore, XG100 has a stronger ability to decompose starch and macromolecular carbohydrates. Secondly, XG100 also showed absolute superiority in its number of glycosyltransferases and carbolytic enzymes, with 8171 and 4555 enzymes, accounting for 69.1% and 85.25% of all enzymes.

Glycosyltransferases are mainly involved in the synthesis of carbohydrates in wine [35], so there are more microorganisms producing polysaccharides in XG100, which is conducive to the formation of functional carbohydrates in fermented wine, which is an important guarantee for brewing high-quality cooking wine, while esters are important flavor compounds in fermented wine and carbohydrate esterases have the function of synthesizing or hydrolyzing esters. It has been reported that the sugar esterase in the fermentation of Japanese sake is related to Jiuqu mold [36], which is conducive to the formation of flavor compounds in cooking wine. AAs, including oxidases and reductases, catalyze redox interactions between species [37]. XG100 had the lowest number of AA genes, indicating that its oxidase and reductase content was low, which inferred that it had a small number of aerobic bacteria and had an advantage in closed fermentation systems.

The SEED Subsystem is an internationally renowned functional classification database and the default database for RAST (Rapid Annotation using Subsystem Technology). As shown in Figure 4B, the top 10 gene modules with the highest abundances were selected in the first-level SEED functional annotation, which were protein metabolism, carbohydrates, amino acids and their derivatives, clustering-based subsystems, the cell wall, the cell wall membrane and capsule, RNA metabolism, adjuvants, vitamins, prosthetic groups, pigments, DNA metabolism, nucleosides and nucleotides, stress responses, and so on.

From a macro point of view, XG100 had the highest relative abundance of this type of gene module, accounting for more than 20% of its genes, which is much larger than that of PTMQ, XG120, and XG200 (Figure 4B). The microscopic analysis showed that protein metabolism accounted for the highest proportion of the four Jiuqu gene types, with it accounting for 0.945%, 2.86%, 1.09%, and 1.071% of PTMQ, XG100, XG120, and XG200, respectively. The overall abundance of carbohydrates was 0.248%, 2.91%, 0.536%, and 1.404%, respectively. The third gene module was amino acids and their derivatives, with the four Jiuqu having 0.269%, 2.097%, 0.452%, and 1.211% abundances, respectively. Their abundance in XG100 was the highest of all three functional genes, which was related to the ability of the *Lichtheimia ramosa* transverse to produce α-amylase, cellulase, protease, saccharification enzymes, and esterase, which was consistent with the highest *Lichtheimia ramosa* abundance in XG100 found in our previous study [19].

These genes are directly related to the taste, texture, and flavor quality of finished wine, so the abundance of such genes can indirectly indicate the quality of Jiuqu. In addition, XG100 also has a high abundance and uniformity of functional genes related to excipients, vitamins, repair groups, pigments, cell walls, and cell membranes, which play an important role in the growth and reproduction of microorganisms and ensure the smooth progress of the fermentation process.

### 3.5. Analysis of the Volatility of Jiuqu with Different Amounts of Shiitake Mushrooms 

#### 3.5.1. GC-IMS Spectral Analysis of Jiuqu’s Raw Materials

**Figure 5:**
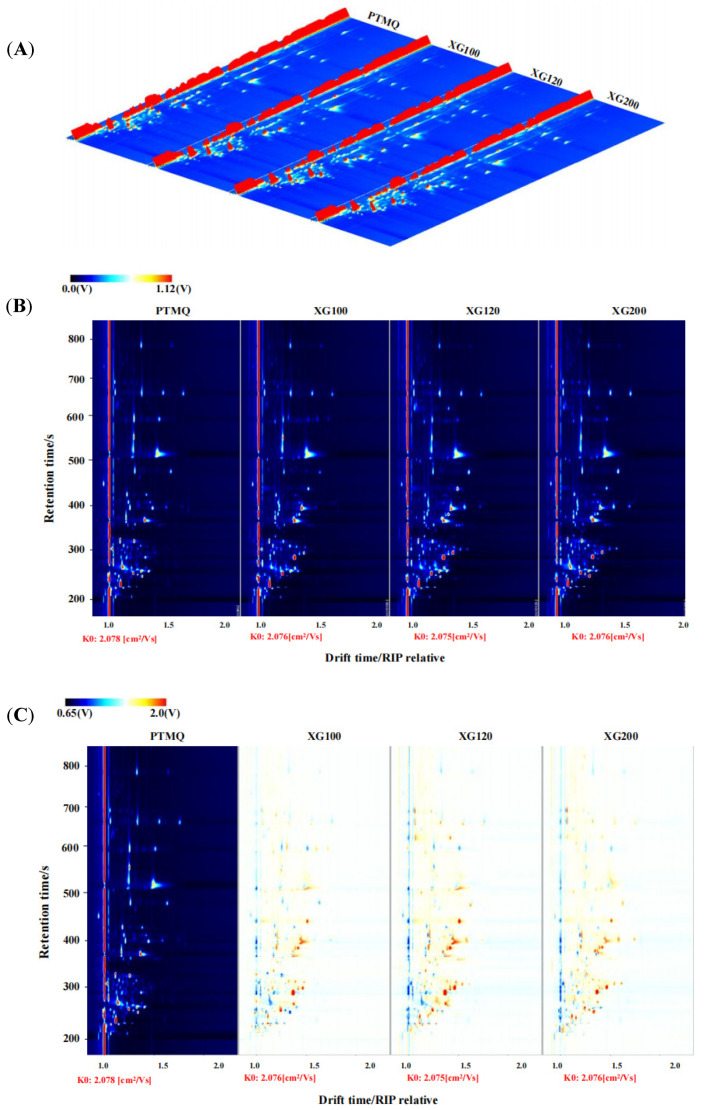
Three-dimensional spectra (**A**) and two-dimensional spectra (**B**,**C**) of volatile substances in Jiuqu samples based on GC-IMS.

Figure 5A is a three-dimensional spectrum obtained by detecting the volatile flavor compounds in different Jiuqu raw materials using GC-IMS technology. The *X* axis represents the drift time, the *Y* axis represents the retention time, and the *Z* axis represents the peak intensity, and each point in the spectrum represents a specific compound, while a compound may have two or more spots representing its dimers or multimers. The content of the odor component is expressed by the shade of a color; the darker the color, the higher the content of the substance. This three-dimensional spectrum represents the flavor compounds of PTMQ, XG100, XG120, and XG200, from left to right (Figure 5A).

In order to better distinguish the differences of the volatile flavor compounds in different Jiuqu raw materials, the three-dimensional spectrum in Figure 5A was converted into a two-dimensional plane map (Figure 5B) and a comparison chart (Figure 5C), obtained by deducting the same part of the PTMQ, so as to further distinguish the volatile flavor compounds in different Jiuqu raw materials. As can be seen from Figure 5B,C, GC-IMS can separate different volatile flavor compounds in different whole Jiuqu raw materials, while the content of most flavor substances fluctuates. This may be due to the change in the content of flavor compounds because of the difference in the amount of shiitake mushrooms added. At the same time, there are relatively more red dots in the shiitake mushroom Jiuqu than in PTMQ (Figure 5C), indicating that it has relatively more types and higher contents of flavor substances.

#### 3.5.2. Analysis of Volatile Flavor Compounds in Jiuqu Raw Materials

The volatile components were qualitatively analyzed based on their gas chromatographic retention time and ion mobility time and matched to the GC-IMS database, and the results are shown in Table 2. As can be seen from Table 2, a total of 79 volatile monomers and dimers of flavor substances were detected from different Jiuqu raw materials, including 12 aldehydes, 19 ketones, 13 alcohols, 19 esters, 4 olefins, 1 acid, 3 ethers, 4 furans, 1 pyrazine, 1 pyridine, 1 triethylamine, and 1 thiazole. The total contents of flavor compounds in PTMQ, XG100, XG120, and XG200 were 4150.6, 6604.31, 7196.97 and 6474.43 μg/100 g, respectively. The most abundant volatile flavor compounds in PTMQ were esters, alcohols, and ketones, while ketones, alcohols, and esters were the most abundant volatile flavor compounds in *Lentinula edodes*, indicating that the inclusion of *Lentinula edodes* greatly increased these flavor profiles. A similar study of Boletus Edulis’ incorporation on volatile flavor profiles was also reported by Guo et al. [37].

From Table 2, it can be concluded that, among the aldehyde compounds, XG120’s content was the highest, followed by XG200 and XG100, and PTMQ’s content was the lowest, accounting for 6.05%, 4.96%, 4.78% and 3.42% of their total Jiuqu raw materials, respectively. Among them, 3-methylbutyraldehyde and benzaldehyde have larger flavor contributions. 3-methylbutyraldehyde has a malt aroma and a roasted nut odor, and its content accounts for 17.69% of the total amount of the four Jiuqu aldehydes, which contribute greatly to the flavor of Jiuqu’s raw materials. Benzaldehyde accounts for 16.04% of the total aldehydes and it has a special almond flavor and a certain effect on the composition of the wine’s flavor [38]. At the same time, 3-methylbutyraldehyde and benzaldehyde were the main volatile flavors in *Lentinula edodes*, so the content of the three types of *Lentinula edodes* Jiuqu was higher than that in PTMQ.

Among the ketones, XG120’s were the highest, followed by XG100 and XG200, and PTMQ’s were the lowest, accounting for 31.7%, 31%, 26.49%, and 22.26% of their total raw materials for Jiuqu, respectively. Ketones can be produced by the oxidative degradation of linoleic acid to hydroperoxides, so the differences in the ketones between different levels of shiitake additions may be caused by the differences in primary metabolites between the levels of mushroom added [39]. Ketones have a characteristic aroma, and short-chain ketones often have fatty and burnt odors [40]. They mainly include 2-propanone (28.18%), 3-pentanone (14.58%), 4-methyl-3-penten-2-one (12.7%), and 2-butanone (11.24%), of which 4-methyl-3-penten-2-one has a honey-like aroma and 2-butanone has a pleasant aroma. It is worth noting that the content of 3-octanone in PTMQ is quite different from that of the other three types of lentinan Jiuqu, which may be due to the fact that this eight-carbon compound is the most important volatile flavor substance in *Lentinula edolets*, and the introduction of *Lentinula edodes* in the Jiuqu increases its content of 3-octanone.

Alcohols are the precursors of ester compounds, which are mainly formed by the conversion of sugars under aerobic conditions and the conversion of amino acids under anaerobic conditions during fermentation [41]. XG200 had the highest content of alcohols, followed by XG120 and XG100, and the PTMQ’s content was relatively low, accounting for 24.48%, 21.48%, 23.28%, and 23.5% of the flavor of their Jiuqu raw materials, respectively. Among the alcohols, 3-methylbutan-1-ol (38.9%) and 2-methyl-1-propanol (19.25%) contributed more to the flavor of the Jiuqu raw materials, and both of them had the smell of malt aroma and roasted nuts. Among the alcohols, 1-octen-3-ol and (E)-2-octen-1-ol both have a particular mushroom flavor [42] and are typical volatile flavor compounds in *Lentinula edodes*. Their contents in shiitake mushroom Jiuqu and common barley Jiuqu are quite different, and, as a typical flavor compound of edible mushrooms, this substance not only provides their characteristic flavor, but also promotes the synthesis of other flavor compounds [42].

Esters can be formed from the combination of alcohols and free fatty acids produced during the oxidation of fats [43]. In addition to imparting a special “floral and fruity aroma” to food [44], they can also mask the “unpleasant and irritating taste” caused by free fatty acids. Among the four types of Jiuqu, the XG200 had the highest content of esters, followed by XG120 and XG100, and their content in PTMQ was the lowest; 23.54%, 19.92%, 18.86%, and 25.06%, respectively. They mainly include (Z)-3-hexenyl butyrate (25.92%), ethyl propanoate (18.76%), and methyl 2-furoate (16.25%). Among them, (Z)-3-hexenyl butyrate has the green aroma of fresh fruit, a slightly creamy aroma; ethyl propanoate has a fruity aroma; and methyl 2-furoate has a pleasant odor, all of which contributed greatly to the flavor of Jiuqu raw materials.

Compared with saturated hydrocarbons, olefins have a lower threshold of terpenes and have floral and fruity aromas, which contribute more to the overall flavor of food [45]. alpha-phellandrene, alpha-pinene, beta-pinene, and delta 3-carene were detected in the four types of Jiuqu samples (Table 2). Alpha-phellandrene has aromas similar to black pepper and mint, alpha-pinene and delta 3-carene have pine-like aromas, and beta-pinene has pine-resin-like aromas.

A total of two kinds of thioethers were detected in the raw materials of Jiuqu, namely diethyl disulfide and dimethyl disulfide, of which dimethyl disulfide has a meaty aroma and onion-like aroma, which is a unique flavor substance of *Lentinula edodes*. Anisole has a pleasant fennel-like aroma. Therefore, the sulfide content of *Lentinula edodes* Jiuqu was significantly increased after the introduction of *Lentinula edodes* into traditional Jiuqu.

Pyrazine compounds are trace compounds that contribute to the formation of liquor flavor and are also important functional components in liquor [46], which mainly exhibit aromas such as chocolate, nut, and peanut, and are considered to be the main source of burnt flavors and baking flavors in Daqu [47]. 2,6-dimethylpyrazine has aromas such as roasted cocoa and peanuts. Pyrazine is mainly formed by the Maillard reaction and is found in a variety of foods, including edible fungi [48]. In addition to pyrazines, furans are also unique flavor components of edible fungi and are also important aromatic substances and characteristic compounds of liquor; a total of four furans were detected in the raw materials of Jiuqu, namely 2-butylfuran, 2-ethylfuran, 2-amylfuran, and tetrahydrofuran. Among them, 2-pentylfuran has been reported in studies as being related to the flavor of edible fungi [49,50]. Among its other compounds, XG100 had the lowest triethylamine content, which may be related to its metabolism of amino acids and their derivatives in the SEED functional annotation above.

### 3.6. Correlation Analysis between the Volatile Flavor Components and Major Microbial Genera in Jiuqu

**Figure 6:**
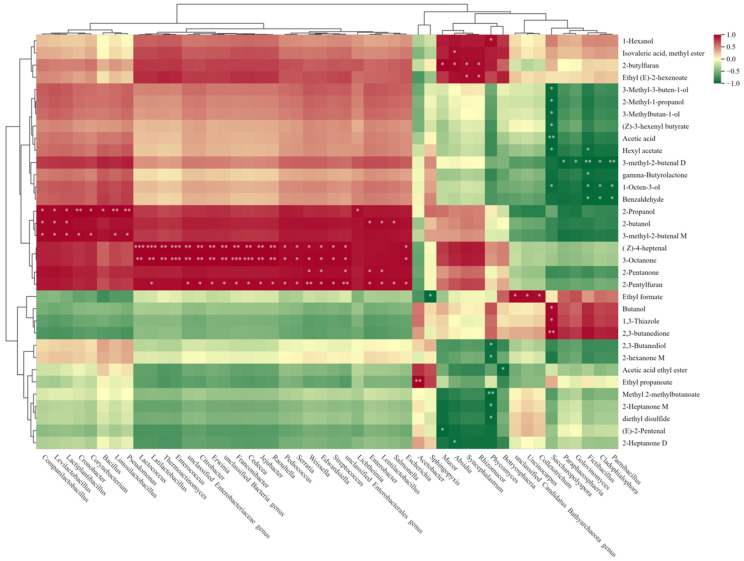
Correlation analysis heat map of microbial communities and volatile flavor compounds in Jiuqu samples. * denotes significant correlation at the 0.05 level; ** denotes significant correlation at the 0.01 level; *** denotes significant correlation at the 0.001 level.

In order to further study the correlation between microorganisms and volatile flavor compounds in the four Jiuqu, the top 100 most abundant microorganisms at the genus level of the four Jiuqu were correlated with their volatile flavor compounds (Figure 6), indicating that the difference in the microbial community diversity of the four Jiuqu was one of the important reasons for the differences in their flavor components. As can be seen in Figure 6, the common microbial genera that are more closely related to the volatile flavor compounds in Jiuqu are *Cronobacter*, *Lactococcus*, *Pediococcus*, *Weisnella*, *Bacillus*, *Enterobacteriaceae*, *Rhizopus*, *Rhizopodium*, Sycophyllum, and *Mucormyces*. (Z)-4-Heptenal and 3-octanone were significantly positively correlated with *Lactococcus*, *Enterococci*, *Citrobacter*, *Erwinella,* and *Raoulella* (*p* < 0.01) and positively correlated with *Pediococcus*, *Serratia*, *Weisnellella*, *Streptococcus,* and unclassified *Enterobacteriaceae* (*p* < 0.05). Among these compounds, 3-octanone has the aromas of green, wax, vegetable, mushroom, cheese, and fruit, which is a typical volatile flavor substance in shiitake mushrooms, while (Z)-4-heptenal has a grassy and oily aroma and a creamy fragrance after dilution. And both of them are the main characteristic aroma substances of XG100.

*Citrobacter*, *Erwinia*, *Raoultella*, *Pediococcus*, *Serratia,* and *Latilactobacillus* were also positively correlated with 2-pentylfuran (*p* < 0.05) and were significantly positively correlated with *Weissella* and unclassified *Enterobacteriaceae* (*p* < 0.01), while 2-butylfuran was positively correlated with *Mucor*, *Absidia*, *Syncephalastrum*, and *Rhizomucor* (*p* < 0.05) (Figure 6). The effects of *Bacillus* and *Lactobacillus* on furans were promotional, which indicated that these microorganisms had a great relationship with the production of furans in Jiuqu, which was consistent with the higher ranking of *Lactobacillus* in its microbial abundance and the higher content of furans in *Lentinula edodes* Jiuqu. 2-propanol was significantly correlated with *Lactiplantibacillus*, *Cronobacter*, *Bacillus*, *Pseudomonas,* and *Limosilactobacillus* (*p* < 0.05). Most of the alcohols and esters were positively correlated with *Weissella* and *Streptococcus*, and studies have shown that *Weissella* has a strong antagonistic effect on these undesirable microorganisms, which can enhance the production of alcohols to inhibit the growth of these pathogenic bacteria [51]. On the other hand, *Streptococcus* has the effect of enhancing esters, which has a positive impact on improving the wine yield and producing high-quality wine. *Saccharopolyspora*, *Pseudobacillus*, and *Bacillus* had certain inhibitory effects on most of the volatile flavor compounds in Jiuqu, among which 1-octen-3-ol, benzaldehyde, and 3-methyl-2-butenal were significantly inhibited (*p* < 0.05). Most of the microorganisms in the four Jiuqu had a promotional effect on 1-hexanol, isovaleric acid (methyl ester), and ethyl (E)-2-hexenoate.

## 4. Conclusions

In summary, the microbial community and volatile flavor compounds of Jiuqu with different levels of *lentinus edodes* were characterized. The results showed that the richness and uniformity of the microbial community composition of Jiuqu were highest in XG100. A total of 1593 genera and 5507 species were identified in the four Jiuqu, and XG100 had the most annotated microbial species, which was consistent with the alpha diversity results of its species. *Aspergillus* and *Paecilomyces* were the most abundant microorganisms at the genus level. XG100 also showed a higher degree of uniformity and the best advantage in its brewing. 

A total of 79 volatile compounds were detected across the four types of Jiuqu samples. The correlation analysis results showed that *Citrobacter*, *Erwinia*, *Raoultella*, *Pediococcus*, *Serratia,* and *Latilactobacillus* were positively correlated with 2-pentylfuran (*p* < 0.05) and were significantly positively correlated with *Weissella* (*p* < 0.01), while 2-butylfuran was positively correlated with *Mucor*, *Absidia*, *Syncephalastrum*, and *Rhizomucor* (*p* < 0.05). 2-propanol was correlated with the appearance *Lactiplantibacillus*, *Cronobacter*, *Bacillus*, *Pseudomonas* and *Limosilactobacillus* (*p* < 0.05). Overall, XG100 displayed comprehensive advantages out of the four Jiuqu samples. This study revealed the microbial community structure and flavor substances of Jiuqu with different amounts of *Lentinula edodes* added, which can provide a reference for the selection of fermentation starters of *Lentinula edodes* Huangjiu in the Qinba Mountain area. Further work on the quality and aroma profiles of the Huangjiu in XG100 will be reported elsewhere.

## Figures and Tables

**Table 1:** Alpha diversity indices of different Jiuqu samples.

Samples	Chao1	ACE	Shannon	InvSimpson	Coverage (%)
PTMQ	338,286.2	348,083.1	9.566496	4928.354	0.9997971
XG100	1,002,053.1	999,030.8	11.874803	19,367.516	0.9999484
XG120	884,536.9	894,061.7	11.294602	19,353.592	0.9999483
XG200	468,667	489,342.9	9.723588	7680.972	0.9998698

Note: PTMQ denotes Jiuqu without shiitake mushrooms’ incorporation. XG100, XG120, and XG200 represent Jiuqu with shiitake mushroom incorporation rates of 2.5%, 3%, and 5%, respectively.

**Table 2:** Types and contents of volatile flavor compounds in Jiuqu raw materials.

NO	Chemicals	CAS	Relative Content (μg/100 g)			
			PTMQ	XG100	XG120	XG200
1	(Z)-4-heptenal	6728-31-0	4.41 ± 0.45 b	24.09 ± 0.63 a	5.25 ± 0.4 b	5.24 ± 0.76 b
2	(E)-2-pentenal	1576-87-0	6.57 ± 0.31 b	4.57 ± 0.54 c	12.49 ± 1.09 a	10.68 ± 0.2 a
3	(Z)-2-methylpent-2-enal	123-15-9	1.78 ± 0.04 d	52.36 ± 1.18 b	98.09 ± 1.31 a	42.16 ± 3.33 c
4	2-methyl propanal	78-84-2	6.22 ± 0.65 c	6.40 ± 0.12 c	16.05 ± 0.24 a	9.27 ± 0.08 b
5	3-methyl butanal	590-86-3	30.31 ± 0.24 c	54.64 ± 0.99 b	82.77 ± 0.47 a	47.09 ± 0.69 b
6	3-methyl-2-butenal M	107-86-8	9.39 ± 0.34 d	17.15 ± 0.91 a	12.18 ± 0.24 c	14.95 ± 1.17 b
7	3-methyl-2-butenal D	107-86-8	3.94 ± 0.08 d	13.05 ± 0.38 a	9.35 ± 0.39 c	11.81 ± 0.61 b
8	menzaldehyde	100-52-7	22.50 ± 0.75 c	52.97 ± 5.18 b	50.71 ± 0.41 b	68.59 ± 4.18 a
9	butyraldehyde	123-72-8	19.64 ± 0.18 c	9.57 ± 0.77 d	29.73 ± 0.85 b	45.34 ± 0.57 a
10	hexanal	66-25-1	3.75 ± 0.42 b	4.76 ± 0.45 b	10.76 ± 0.52 a	3.24 ± 0.38 b
11	pentanal	110-62-3	6.69 ± 0.21 d	36.72 ± 1.39 b	57.47 ± 1.37 a	26.01 ± 0.34 c
12	propanal	123-38-6	26.83 ± 0.61 c	39.67 ± 2.05 b	50.35 ± 2.2 a	36.93 ± 0.09 b
Total aldehydes			142.02 ± 3.17 c	315.95 ± 4.09 b	435.20 ± 3.38 a	321.31 ± 5.62 b
13	1-hydroxy-2-propanone	116-09-6	5.35 ± 0.65 b	8.45 ± 0.29 b	15.86 ± 1.83 a	11.96 ± 1.61 a
14	2,3-butanedione	431-03-8	33.18 ± 0.56 a	9.08 ± 0.92 c	10.90 ± 0.93 b	10.76 ± 0.17 b
15	2-butanone M	78-93-3	20.69 ± 0.45 b	29.40 ± 1.62 a	20.14 ± 0.47 b	13.63 ± 0.58 c
16	2-butanone D	78-93-3	94.00 ± 0.24 d	288.20 ± 6.91 a	231.04 ± 0.97 b	169.30 ± 2.02 c
17	2-heptanone M	110-43-0	70.98 ± 0.91 c	68.00 ± 0.97 d	97.14 ± 0.53 a	91.32 ± 0.69 b
18	2-heptanone D	110-43-0	38.89 ± 1.88 c	28.35 ± 0.29 d	67.40 ± 1.13 b	72.70 ± 3.28 a
19	2-hexanone M	591-78-6	15.54 ± 0.69 c	21.15 ± 0.84 b	27.36 ± 0.31 a	26.73 ± 1.16 a
20	2-hexanone D	591-78-6	5.64 ± 0.78 d	7.40 ± 0.38 c	19.57 ± 0.37 a	13.55 ± 0.68 b
21	2-methyltetrahydrofuran-3-one	3188-00-9	32.99 ± 0.73 c	31.67 ± 1.46 c	89.84 ± 2.06 a	62.00 ± 1.12 b
22	2-octanone	111-13-7	40.85 ± 6.55 d	46.67 ± 4.51 c	56.18 ± 5.6 b	63.00 ± 10.46 a
23	2-pentanone	107-87-9	26.44 ± 0.78 c	54.90 ± 0.44 a	38.40 ± 0.75 b	38.54 ± 1.48 b
24	2-propanone	67-64-1	334.45 ± 1.33 d	585.79 ± 9.84 a	563.37 ± 0.99 b	479.24 ± 2.53 c
25	3-hydroxy-2-butanone	513-86-0	49.91 ± 0.74 c	71.52 ± 1.96 a	68.14 ± 2.08 a	62.36 ± 2.64 b
26	3-methyl-2-pentanone M	565-61-7	13.92 ± 1.11 d	39.26 ± 0.49 a	37.35 ± 0.06 b	30.10 ± 0.17 c
27	3-methyl-2-pentanone D	565-61-7	12.69 ± 0.78 d	72.66 ± 1.61 c	162.23 ± 1.65 a	104.96 ± 0.73 b
28	3-octanone	106-68-3	6.94 ± 1.46 c	38.90 ± 0.97 ab	39.21 ± 0.05 a	38.59 ± 1.3 b
29	3-pentanone	96-22-0	45.63 ± 2.79 d	339.28 ± 2.59 b	392.66 ± 3.51 a	208.10 ± 1.86 c
30	4-methyl-3-penten-2-one	141-79-7	75.63 ± 4.43 d	284.50 ± 4.68 b	319.83 ± 2.83 a	204.80 ± 4.94 c
31	4-methyl-2-pentanone	108-10-1	2.23 ± 0.52 d	18.78 ± 0.19 b	25.11 ± 1.13 a	10.83 ± 0.67 c
Total ketones			925.97 ± 7.15 d	2043.96 ± 9.36 b	2281.73 ± 15.7 a	1712.47 ± 13.4 c
32	(E)-2-octen-1-ol	18409-17-1	38.86 ± 11.47 d	110.43 ± 20.85 c	161.93 ± 11 b	217.18 ± 5.71 a
33	1-hexanol	111-27-3	13.11 ± 1.3 a	14.69 ± 0.31 a	10.01 ± 0.56 b	9.53 ± 0.41 b
34	1-octen-3-ol	3391-86-4	46.62 ± 11.18 c	78.78 ± 2.13 b	75.43 ± 5.97 b	86.91 ± 6.44 a
35	1-pentanol	71-41-0	48.17 ± 1.78 a	26.93 ± 1.5 c	25.04 ± 1.07 c	33.11 ± 1.49 b
36	2,3-butanediol	513-85-9	5.99 ± 0.06 c	20.10 ± 5.96 c	42.97 ± 5.73 b	52.70 ± 8.68 a
37	2-butanol	78-92-2	17.10 ± 0.41 d	45.24 ± 1.34 a	26.01 ± 0.33 c	31.14 ± 0.81 b
38	2-methyl-1-propanol	78-83-1	181.73 ± 5.54 d	301.36 ± 2.58 b	312.77 ± 1.05 a	290.33 ± 2.63 c
39	2-propanol	67-63-0	35.17 ± 4.38 c	59.84 ± 7.01 a	36.19 ± 1.07 c	52.01 ± 3.03 b
40	3-heptanol	589-82-2	86.92 ± 2.72 d	113.73 ± 1.85 c	126.54 ± 3.27 a	118.87 ± 0.44 b
41	3-methyl-3-buten-1-ol	763-32-6	11.66 ± 0.48 c	27.87 ± 1.21 a	26.61 ± 0.32 a	23.11 ± 0.54 b
42	3-methylbutan-1-ol	123-51-3	387.63 ± 6.08 d	638.83 ± 7.05 a	600.15 ± 4.84 b	568.83 ± 2.63 c
43	butanol	71-36-3	19.33 ± 0.23 a	13.01 ± 0.19 b	9.58 ± 0.36 d	11.11 ± 0.41 c
44	propanol	71-23-8	85.39 ± 5.19 bc	84.53 ± 0.63 c	92.62 ± 0.22 a	87.98 ± 1.29 b
Total alcohols			977.67 ± 9.18 d	1535.35 ± 15.3 c	1545.83 ± 10.2 b	1582.81 ± 9.1 a
45	2-methylbutanoic acid ethyl ester	7452-79-1	10.23 ± 0.61 c	15.98 ± 0.46 b	20.64 ± 0.28 a	17.61 ± 0.55 b
46	2-methylpropyl acetate	110-19-0	2.92 ± 0.44 c	3.54 ± 0.22 c	12.80 ± 1.04 b	19.46 ± 0.98 a
47	3-methylbutyl2-methylbutanoate	27625-35-0	3.67 ± 0.8 d	6.55 ± 0.27 c	28.60 ± 1.63 a	13.23 ± 1.1 b
48	acetic acid ethyl ester	141-78-6	52.82 ± 5.09 c	15.64 ± 1.52 d	55.79 ± 2.37 b	100.43 ± 1.21 a
49	ethyl (E)-2-butenoate	623-70-1	52.60 ± 1.28 a	50.69 ± 1.77 b	40.29 ± 1.22 d	44.96 ± 1.94 c
50	ethyl (E)-2-hexenoate	27829-72-7	61.61 ± 1.12 b	73.40 ± 0.81 a	55.83 ± 2.59 c	51.27 ± 1.97 d
51	ethyl 3-methylbutanoate	108-64-5	74.55 ± 1.19 b	49.77 ± 0.82 d	70.96 ± 1.92 c	95.47 ± 1.94 a
52	ethyl formate	109-94-4	37.15 ± 1.01 b	35.56 ± 2.25 c	40.28 ± 1.05 a	29.11 ± 0.48 d
53	ethyl propanoate	105-37-3	265.87 ± 4.36 b	208.02 ± 5.67 d	215.30 ± 1.12 c	294.24 ± 6.31 a
54	hexyl acetate	142-92-7	17.52 ± 1.11 c	33.92 ± 1.85 ab	31.57 ± 0.43 b	35.89 ± 1.01 a
55	isovaleric acid, methyl ester	556-24-1	14.62 ± 1.11 b	18.42 ± 1.05 a	13.32 ± 0.25 bc	12.68 ± 0.62 c
56	methyl 2-furoate	611-13-2	62.30 ± 10.84 d	238.42 ± 20.01 c	270.80 ± 10.71 b	280.35 ± 19.47 a
57	methyl 2-methylbutanoate	868-57-5	2.47 ± 0.59 d	5.24 ± 0.63 c	27.55 ± 0.61 a	22.66 ± 0.46 b
58	methyl acetate	79-20-9	10.65 ± 1.71 c	18.21 ± 0.52 b	42.98 ± 0.64 a	20.23 ± 0.35 b
59	propanoic acid propyl ester	106-36-5	2.29 ± 0.56 d	13.57 ± 0.52 b	15.06 ± 0.42 a	8.75 ± 0.07 c
60	propyl butanoate	105-66-8	23.47 ± 1.13 c	27.26 ± 1.13 b	38.13 ± 1.16 a	23.95 ± 0.67 c
61	(Z)-3-hexenyl butyrate	16491-36-4	292.30 ± 5.91 d	355.85 ± 25.9 b	364.14 ± 11.23 a	346.53 ± 17.28 c
62	gamma-butyrolactone	96-48-0	48.18 ± 2.31 d	67.71 ± 1.39 c	69.98 ± 2.55 b	76.03 ± 8.75 a
63	1-butanol, 3-methyl-, acetate	123-92-2	7.41 ± 0.6 c	5.89 ± 0.53 d	19.44 ± 1.24 b	28.95 ± 1.07 a
Total esters			1042.64 ± 20.89 d	1243.64 ± 17.37 c	1433.47 ± 15.63 b	1521.80 ± 17.11 a
64	alpha-phellandrene	99-83-2	48.59 ± 1.1 d	77.83 ± 2.48 a	72.10 ± 2.43 b	65.57 ± 2.17 c
65	alpha-pinene	80-56-8	9.44 ± 0.14 b	5.25 ± 0.75 c	9.98 ± 0.94 b	12.94 ± 1.5 a
66	beta-pinene	127-91-3	7.38 ± 0.91 d	45.08 ± 3.28 b	54.84 ± 0.45 a	35.31 ± 1.35 c
67	delta 3-carene	13466-78-9	32.42 ± 2.01 d	53.59 ± 3.86 a	42.29 ± 3.28 b	36.72 ± 1.7 c
Total olefins			97.83 ± 2.09 c	181.75 ± 4.18 a	179.21 ± 3.47 a	150.54 ± 2.93 b
68	acetic acid	64-19-7	274.89 ± 16.72 d	411.87 ± 11.25 c	439.26 ± 19.33 a	425.32 ± 5.99 b
Total acids			274.89 ± 16.72 d	411.87 ± 11.25 c	439.26 ± 19.33 a	425.32 ± 5.99 b
69	diethyl disulfide	110-81-6	3.85 ± 1.11 c	3.79 ± 0.22 c	18.86 ± 0.54 a	13.88 ± 0.96 b
70	dimethyl disulfide	624-92-0	28.51 ± 1.41 d	48.15 ± 1.03 a	36.84 ± 0.66 b	31.14 ± 0.19 c
71	anisole	100-66-3	51.23 ± 1.68 d	60.69 ± 1.11 c	75.68 ± 3.2 a	64.16 ± 2.61 b
Total ethers			83.60 ± 2.98 d	112.62 ± 2.14 b	131.38 ± 2.78 a	109.18 ± 1.89 c
72	2-butylfuran	4466-24-4	116.27 ± 4.48 b	131.26 ± 4.87 a	97.74 ± 2.64 d	100.65 ± 0.57 c
73	2-ethyl furan	3208-16-0	57.28 ± 1.41 d	96.12 ± 1.45 b	127.77 ± 1.05 a	71.17 ± 0.65 c
74	2-pentylfuran	3777-69-3	98.73 ± 1.13 d	153.99 ± 1.03 a	115.32 ± 1.84 b	110.86 ± 2.27 c
75	tetrahydrofuran	109-99-9	9.24 ± 1.11 d	17.73 ± 0.22 c	30.35 ± 0.66 a	20.58 ± 0.19 b
Total furans			281.52 ± 4.71 d	399.10 ± 4.39 a	371.18 ± 3.15 b	303.26 ± 2.93 c
76	(2,6)-dimethylpyrazine	108-50-9	41.19 ± 2.54 d	58.54 ± 3.01 c	68.13 ± 1.68 a	65.88 ± 3.39 b
77	pyridine	110-86-1	163.30 ± 3.42 d	197.62 ± 5.78 a	191.38 ± 3.6 b	169.08 ± 3.81 c
78	triethylamine	121-44-8	19.05 ± 2.25 b	17.03 ± 1.8 c	31.34 ± 1.33 a	21.25 ± 0.68 b
79	1,3-thiazole	288-47-1	100.92 ± 1.99 a	86.88 ± 3.41 d	88.87 ± 1.79 c	91.54 ± 2.45 b
Other components			324.46 ± 4.98 d	360.08 ± 6.13 b	379.71 ± 5.26 a	347.75 ± 6.11 c
Total volatile substances			4150.60 ± 63.98 d	6604.31 ± 72.45 b	7196.97 ± 79.81 a	6474.43 ± 75.46 c

Notes: volatile flavor substance suffixes D and M denote dimers and monomers, respectively. Different letters in the same row denote significant differences (*p* < 0.05).

## Data Availability

The original contributions presented in the study are included in the article, further inquiries can be directed to the corresponding author.
